# Unravelling Influence Factors in Pattern Recognition Myoelectric Control Systems: The Impact of Limb Positions and Electrode Shifts

**DOI:** 10.3390/s24154840

**Published:** 2024-07-25

**Authors:** Bingbin Wang, Jinglin Li, Levi Hargrove, Ernest Nlandu Kamavuako

**Affiliations:** 1 Department of Engineering, King′s College London, London WC2R 2LS, UK; bingbin.wang@kcl.ac.uk (B.W.);; 2 Center for Bionic Medicine, Shirley Ryan Ability, Chicago, IL 60611, USA; l-hargrove@northwestern.edu; 3 Department of Physical Medicine and Rehabilitation, Feinberg School of Medicine, Northwestern University, Chicago, IL 60611, USA; 4 Faculté de Médecine, Université de Kindu, Site de Lwama II, Kindu, Maniema, Congo

**Keywords:** changes in feature space, electrode shifts, electromyography, limb positions, myoelectric control, pattern recognition

## Abstract

Pattern recognition (PR)-based myoelectric control systems can naturally provide multifunctional and intuitive control of upper limb prostheses and restore lost limb function, but understanding their robustness remains an open scientific question. This study investigates how limb positions and electrode shifts—two factors that have been suggested to cause classification deterioration—affect classifiers’ performance by quantifying changes in the class distribution using each factor as a class and computing the repeatability and modified separability indices. Ten intact-limb participants took part in the study. Linear discriminant analysis (LDA) was used as the classifier. The results confirmed previous studies that limb positions and electrode shifts deteriorate classification performance (14–21% decrease) with no difference between factors (*p* > 0.05). When considering limb positions and electrode shifts as classes, we could classify them with an accuracy of 96.13 ± 1.44% and 65.40 ± 8.23% for single and all motions, respectively. Testing on five amputees corroborated the above findings. We have demonstrated that each factor introduces changes in the feature space that are statistically new class instances. Thus, the feature space contains two statistically classifiable clusters when the same motion is collected in two different limb positions or electrode shifts. Our results are a step forward in understanding PR schemes’ challenges for myoelectric control of prostheses and further validation needs be conducted on more amputee-related datasets.

## 1. Introduction

Myoelectric control is the state-of-the-art control system for a sophisticated upper-limb prosthetic system. This control scheme reflects the user’s intention and uses the muscle’s electrical signals (electromyogram, EMG) to achieve the desired function. Currently, most commercially available upper-limb prostheses use conventional two-site proportional control, on/off control, and direct control, which maps different amplitudes of EMG signals to corresponding mechanical output [1]. Although traditional myoelectric control schemes can provide reasonable controllability, they have limited functionality that cannot fulfil users’ daily living requirements. According to the prosthetic usage surveys [2,3], there has been no significant decrease in the prosthetic abandonment rate since 2007. Users complained most about the lack of functionality and discomfort.

Machine-learning-based myoelectric control systems are the primary developing trend for prosthetics, where pattern recognition (PR) techniques are widely employed [4] and are now available commercially. PR-based control systems directly decode EMG signals acquired from muscles into different motion patterns and can potentially achieve intuitive control. PR-based control systems perform equivalent to or better than conventional control approaches when used by amputees within their home environment [5]. EMG signals are stochastic and are sensitive to extrinsic factors (e.g., electrode shift [6], skin impendence change [7]) and intrinsic variations (muscle fatigue [8], mutual adaptation [9]). Obtaining identical EMG signals consistently is impossible, even with a fixed setup and movements. These factors are evident in home trials and are mitigated using periodic system recalibration [5]. The performance of PR-based control systems heavily depends on feature extraction and classifications. The change in the EMG signal feature space subsequentially affects the performance. Hence, the influencing factors impose repeated calibration sessions in PR-based control systems.

In the upper-limb prosthesis control, a change in the limb position means using the prosthesis in different arm positions. Amputees need to adjust their limb positions to perform specific daily living activities, such as picking items off the ground or reaching overhead. Different limb positions require corresponding muscle coordination, which generates different EMG signal patterns. In addition, the underlying topography of the muscle fibers and skin may shift relative to the electrode in different limb positions [10]. Fougner et al. [11] demonstrated that limb position variation substantially impacted the myoelectric signal classification performance, where the average classification error increased from 3.8% to 18%. They also showed that the errors in intra-position classification (training and testing in same limb position) were relatively lower than in the inter-position classification (training and testing in different limb positions), which were 3.8% and 21.1%, respectively. Jochumsen et al. [12] compared the effect of limb positions on the surface and intramuscular EMG and showed that both types of EMG were similarly affected by limb positions. The classification accuracies decreased by 12–16% in between-position performance. Due to the requirement of frequently changing limb position for amputees to complete activities of daily living, the mitigation of the effect of limb position on real-time performance is necessary. Teh and Hargrove [13] demonstrated that limb position significantly affected amputees’ and intact-limb subjects’ real-time virtual prosthesis control performance.

Electrode shift usually happens when the user redons the prosthesis, leading to significant variation in the EMG signal amplitude and can be easily confused with a change in muscle activation patterns. The main reason for the variation is related to the innervation zone of each motor unit [10]. According to Rainoldi et al. [14], little disturbances will significantly vary EMG amplitude when electrodes are close to an innervation zone. Young et al. [15] investigated how the size and orientation of electrodes affect the system’s robustness to electrode shift. Based on displacement up to 2 cm, they found that the direction of the shifts parallel to muscle fibers (5–20% error) had a more negligible effect on classification accuracy than the perpendicular shifts (40% error). High-density (HD) EMG electrodes were proposed to mitigate the effect of shifts [16,17]. Nevertheless, the level of performance degradation induced by electrode shifts in HD EMG is sensitive to the density of HD EMG electrodes [18].

For each influencing factor, various approaches have been developed to mitigate performance degradation. Classifiers can potentially learn the underlying characteristics of affected data for each motion, which gives inspiration for extensive training [19,20,21]. Gigli et al. [21] explored a dynamic training protocol to use the EMG signals acquired from a continuous limb movement that contains all motion patterns of interest. The results showed the advantages of the dynamic training protocol, such as satisfying controllability and a less tiresome data collection procedure. The dynamic training protocol also demonstrated a similar level of controllability improvement in amputees [13]. In addition, some features and adaptive machine-learning techniques are less sensitive to the fluctuations of EMG signals [22,23,24,25,26]. Stango et al. [25] extracted spatial features from HD EMG signals to classify nine and seven classes for intact-limb and amputated subjects, respectively. The results indicated that spatial features were less sensitive to electrode shifts (±1 cm) than classic features. Ameri et al. [26] manifested a novel self-recalibrated system based on transfer learning with convolutional neural networks (TL-CNN) to reduce the effect of electrode shifts. They compared the adaptive ability to electrode shifts between the proposed TL-CNN with other state-of-the-art techniques. The results showed that TL-CNN was more effective in reducing errors.

Current studies can achieve promising results in classifying motion patterns under the effect of a single influencing factor. However, several factors could appear simultaneously in the practical use of prostheses. For instance, electrode shifts could happen due to the weight of the prosthesis and gravity while the limb position changes. Asogbon et al. [27] investigated the co-existing impact of mobility of the subject and variation of contraction force on the PR-based prostheses and reduced the factor-induced error by 7.50~17.97% through robust feature extraction. Gu et al. [28] explored robust features and classifiers under electrode shifts, force variation, limb position change and temporary drift. They utilized an adaptive learning method to mitigate performance degradation. In contrast to the laboratory experiment to simulate the potential factors that could occur during the use of the prostheses in daily life, researchers have attempted to monitor the control of at-home prostheses to improve their functionality and performance [5,29] and tried to narrow the gap between academic research and clinical application. They have only investigated how users interact with their prostheses, analyzing the quality of recalibration data and the potential reasons for low-quality EMG signals. Meanwhile, without the support of additional devices or data, the occurrence of influencing factors such as electrode shift sand changes in limb position while using a prosthesis remains unknown. These factors could be the primary causes of recalibration. Hence, it is critical to recognize different influencing factors in EMG signals when performance degradation occurs during daily use to help researchers understand how frequently each factor occurs so that they can adopt the corresponding solution further. 

While the above studies [5,14,15,16,17,18,19,20,21,22,23,24,25,26,27,28,29] have described performance degradation or proposed mitigating solutions, our study focuses on understanding the causes of classifier failure to inform future adaptive approaches. Also, the extent to which influencing factors affect the feature space and class distributions has received less attention in the literature. Hence, this study aims to quantify the effect of limb positions and electrode shifts on classification. We acquired EMG data from four frequently used limb positions in daily life and from four different electrode shifts that could occur during daily use. To further validate our results in practical settings, we also utilized a dataset from amputees involving four different limb positions. We analyzed how feature space was changed under different factors and demonstrated for the first time that these factors generate additional class instances that make the problem challenging to address from purely a machine-learning perspective. 

## 2. Materials and Methods

We used our own dataset recorded in the BioSignals and Sensors Laboratory at King’s College London, as described below, and a previously published dataset, as detailed in [30], to evaluate our approach on amputees.

### 2.1. Participants

A total of 10 intact-limb, right-handed participants (6 males and 4 females, with an age range from 23 to 30 years) participated in the experiment. They were healthy and able to perform all instructed hand motions and limb positions. All experimental procedures were approved by Kings College London’s Research Ethics Board (MRM-21/22-22163). Participants provided written informed consent. 

### 2.2. Experimental Setup

Participants sat comfortably, and the skin over the target muscles was cleaned using alcohol wipes. Six Trigno Avanti Sensor wireless electrodes (Delsys Incorporated, Natick, MA, USA) were attached circumferentially to the participants’ right forearm for surface EMG signal acquisition. Two electrodes were placed on the extensor muscles (extensor carpi ulnaris and extensor radialis), two on the flexor muscles (flexor carpi radialis and flexor carpi ulnaris), and the last two on the pronator and supinator muscles. EMG was analogue filtered between 20 and 500 Hz [31] and sampled at 2148 Hz which was preset in Delsys software (EMGworks^®^ version 4.5.0). 

**Figure 1:**
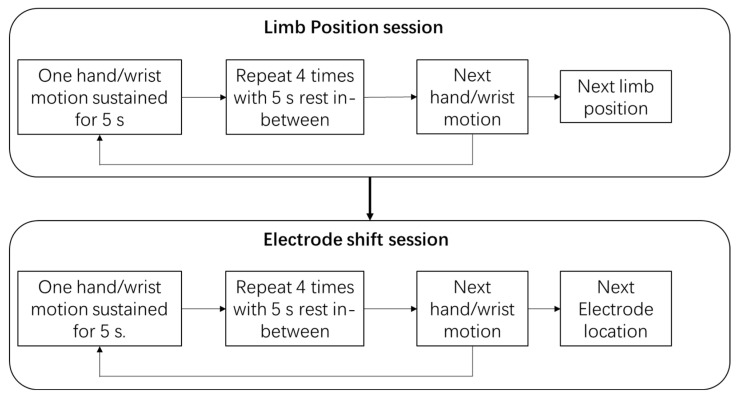
The workflow of the entire experiment.

Eight sessions were recorded for each participant, namely four limb positions and four electrode shifts. In each session, participants performed seven motions in random order: close hand (CH), open hand (OH), wrist flexion (FL), wrist extension (EX), pronation (PN), supination (SN), and rest (RT). Each motion was sustained for up to five seconds and repeated four times consecutively before proceeding to the next motion, with five seconds of rest between repetitions and between motions. The workflow is shown in Figure 1.

#### 2.2.1. Limb Position Sessions

**Figure 2:**
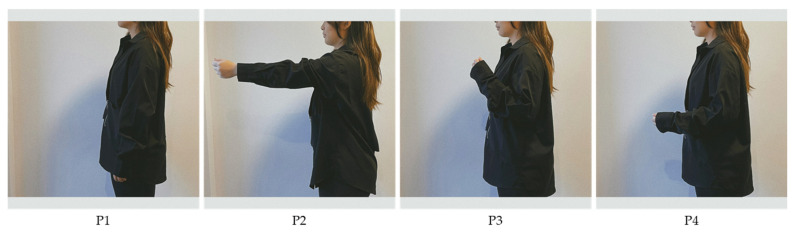
The four limb positions are demonstrated in standing posture. P1, neutral, relaxed position with the arm at the side and neutral wrist; P2, shoulder flexion 90°, elbow extended, neutral wrist; P3, shoulder relaxed, elbow flexion 135°, neutral wrist; P4 shoulder relaxed, elbow flexion 90°, neutral wrist.

Four limb position sessions were conducted on each participant, as shown in Figure 2.

#### 2.2.2. Electrode Shift Sessions

Four electrode shifts were investigated in both transversal and longitudinal directions in each session, which was achieved by changing the placement of the electrode in a small range related to the reference position. To avoid the mixture of limb position and electrode shift and keep other configurations constant, we assumed P1 as the unaffected condition and simulated electrode shifts based on P1. Details of each electrode shift are described in Table 1.

### 2.3. Signal Processing and Feature Extraction

The obtained EMG signals were processed using MATLAB R2022b on the desktop with an AMD Ryzen 7 3700X processor (AMD, San Diego, CA, USA). The recorded EMG signal was digitally filtered by a third-order band-pass Butterworth filter (20 to 500 Hz) and a 50 Hz notch filter to attenuate power line interferences. The filtered signals were segmented afterwards into windows of 200 ms with 50 ms overlaps. Hudgins’s time-domain features (mean absolute value, waveform length, zero-crossing, slop sign change) [32] with Willison amplitude were extracted from each window.

### 2.4. Data Analysis

#### 2.4.1. Between Factors Analysis

Linear discriminant analysis (LDA) was selected for motion classification for each factor. Different classification scenarios were performed, within factor classification (WFC) and between factor classification (BFC). For WFC, the classification accuracies were calculated in the scenario where the training and test data belonged to the same factor using a 4-fold cross-validation procedure. The effect of training the classifier on data from the base factor (P1/S0) and testing on other positions and electrode shifts, respectively, was tested in the BFC scenario. 

A one-way ANOVA was applied to the BFC results to test for significant differences between the classification performance of each level of influencing factor and the corresponding performance degradation.

#### 2.4.2. Feature Space Quantification

Consistency (repeatability index) within a motion and separability between motions were quantified based on the following metrics to investigate how the feature space changed under different influence factors:

- Repeatability index (RI)

The repeatability index, proposed by Bunderson and Kuiken [33], measures how well a subject can reproduce consistent motion patterns. In our case, we found the baseline RI, defined as the RI within the four repetitions of each motion within the same factor, and averaged over factors. Then, we calculated the RI between the four limb positions and the four electrode shifts. For consistency with the baseline RI, we randomly chose one trial from each factor associated with the same motion and calculated the RI such that we also had four trials for limb positions and electrode shifts. A lower RI indicates a higher consistency:

(1) $$RI=\frac{1}{K}\sum_{i=1}^{K}\left(\frac{1}{K}\sum_{j=1}^{K}\frac{1}{2}\sqrt{{\left(\mu_{i}-\mu_{j}\right)}^{T}S_{i}^{-1}\left(\mu_{i}-\mu_{j}\right)}\right)$$

where K is the number of trials, $S_{i}$ and $\mu_{i}$ are the covariance and the mean of trial i (the target trial to be compared), and $\mu_{j}$ is the mean of the feature vector of trial j.

- Modified separability index (mSI)

The separability index, as defined by [33], calculates the distance between the centroid of an ellipse representing each class and its adjacent class. The modified separability index [34,35] is similar to the separability index. It uses the average covariance of the target and the compared distributions. We measured the mSI between each motion under the effect of different factors. The mSI within each factor was used as the baseline. Then, we computed the mSI between factors to quantify the changes. A larger mSI implies greater separability between classes:

(2) $$mSI=\frac{1}{M}\sum_{i=1}^{M}{min\;}_{j=1,\;j\neq i}^{j=M}\frac{1}{2}\sqrt{{\left(\mu_{i}-\mu_{j}\right)}^{T}S^{-1}(\mu_{i}-\mu_{j})}$$

where M is the number of motion classes, $\mu_{i}$ and $\mu_{j}$ are the centroids of $i_{th}$ class and $j_{th}$ class, and S is the average covariance of classes being compared. 

Furthermore, we performed a two-way ANOVA and multi-comparisons (corrected using Bonferroni [36]) to investigate differences in RI while considering the effects of various factors and determining whether those differences were motion-dependent. As no statistically significant difference was found between baseline mSI, we averaged them separately across limb positions and electrode shifts. Subsequently, we conducted a one-way ANOVA to assess whether there were significant differences in mSI between baseline mSI and merged factors mSI. 

#### 2.4.3. Effect of Factors on Class Distributions

The effect of factors on class distributions was quantified, with each factor being set as a separate class instead of using motions as classes, as in Section 2.4.1. We hypothesized that position or electrode movement changes create new class instances that can be classified with high accuracy. As this is a feasibility investigation, LDA was used due to its simplicity and fast processing time. We performed five types of factor classification, as described in Table 2. The first two classification types examined how influencing factors could be classified for single motions, while the last three investigated how influencing factors could be classified using all motions together. For consistency, we also tested each repetition of motions within each factor as a class.

As for statistical analysis, a one-way ANOVA and multi-comparison procedure (corrected using Bonferroni) were conducted on the first two types of classification to investigate if there were significant differences in classification performance between motions.

Furthermore, feature space distributions were represented using principal component analysis. The first two principal components of the feature spaces of factors for all motions and subjects were plotted as a visual representation of class distributions. 

#### 2.4.4. Effect of Limb Position on Class Distributions on Amputees

**Figure 3:**
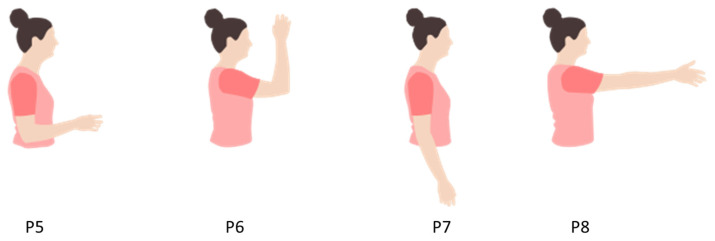
The subjects in [30] performed these four limb positions. P5, shoulder relaxed, elbow flexion 90°, neutral wrist; P6 shoulder flexion 90°, elbow flexion 90°, neutral wrist neutral; P7, relaxed position with the arm at the side and neutral wrist; P8, shoulder flexion 90°, elbow extended, neutral wrist.

To address concerns regarding data dependency, an additional limb position-related dataset was utilized, comprising data from fourteen intact-limb participants and five amputees sourced from [30]. Four limb positions (Figure 3) and seven hand motions (rest, hand open and close, wrist pronation and supination, wrist flexion and extension) were performed by all intact-limb participants and two amputees. Three of the five amputees without experience with pattern recognition could only perform five out of seven gestures (not wrist flexion and extension).

Between factors, analysis was conducted based on the database. Based on limb positions in our self-built data, P7 was assumed to be the unaffected limb position. However, we could not quantify the separability and repeatability of motions based on our metrics in the feature space because each motion was recorded only once. Instead, the plots of the first three principal components were used to visualize the feature space distributions. In addition, influence factor classification Type 1 (only including limb position classes) and Type 3 were performed on intact-limb and amputated subjects’ data. A two-way ANOVA (corrected using Bonferroni) was performed to investigate the differences in influencing factor classification performance between amputees and intact-limb subjects and the corresponding extent of degradation of limb position changes.

## 3. Results

### 3.1. Between Factor Analysis

The WFC accuracy was, on average, over 98% for each level of influence factor, and there were no significant differences between factors. This means the motion patterns were well separated within each influence factor and we could proceed with further analysis.

Table 3 and Table 4 summarize WFC for the base position and BFC accuracies. Limb positions and electrode shifts significantly affected (*p* < 0.05, one-way ANOVA) the BFC. However, the degradations of classification accuracy did not differ substantially between each level of limb positions (*p* = 0.59, one-way ANOVA) and between each level of electrode shifts (*p* = 0.65, one-way ANOVA). 

### 3.2. Feature Space Quantification

**Figure 4:**
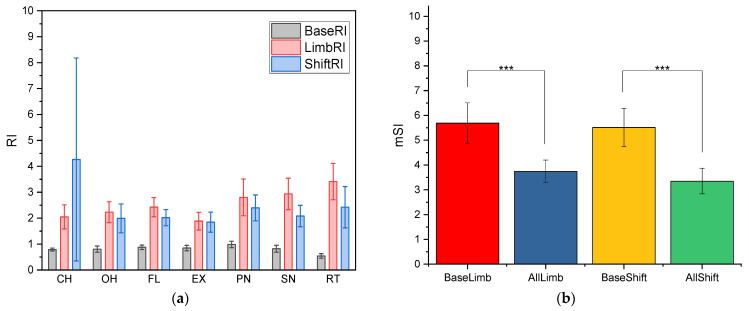
Feature distribution quantified using RI and mSI: (**a**) RI between trials from the same factors and between randomly picked trials from each factor level, and (**b**) mSI between motions for limb position changes and electrode shifts. Significant differences (*p* < 0.001) are denoted by ***.

The results of the mSI and RI are displayed in Figure 4. In Figure 4a, the RIs increased when limb positions and electrode shifts were induced, indicating low consistency between each level of factors. There were significant differences (*p* < 0.05, two-way ANOVA) between the baseline and changes in limb position as well as electrode shifts, but no significant differences (*p* > 0.05, two-way ANOVA) between the two types of factors in RI. The difference did not depend on motions (*p* > 0.05, two-way ANOVA). Regarding separability, mSI of limb positions and electrode shifts decreased significantly from baseline (*p* < 0.05, one-way ANOVA), suggesting the degradation in separability between motions.

### 3.3. Effect of Factors on Class Distributions

The results of all types of classification for each motion are summarized in Table 5. The classification accuracies of single-motion-based classification types (Type 1 and 2) are detailed in Table 6. We can accurately classify different factors using single motions. This performance decreased when all motions were used together, indicating a merge of clusters. The results of the multi-comparison procedure showed that a significant difference only existed between EX and RT. 

To ascertain that the above classification is statistically meaningful, classifying different trials of each influencing factor performed similarly to chance (~50%) or below, indicating no clear separation between repetitions as classes. The results are shown in Table 7.

**Figure 5:**
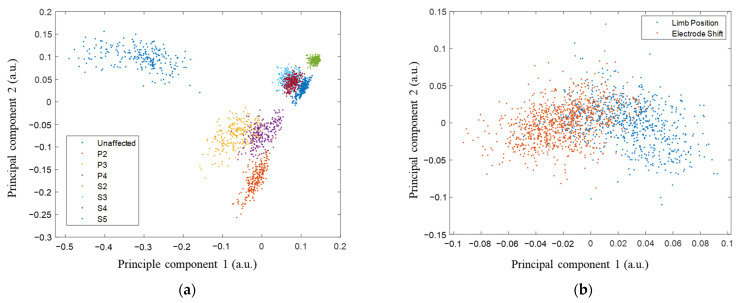
The first two principal components of feature spaces for the influencing factor class are based on single motion: (**a**) the distribution of each level of limb position and electrode shift in CH, and (**b**) the distribution of electrode shifts and limb positions in RT.

**Figure 6:**
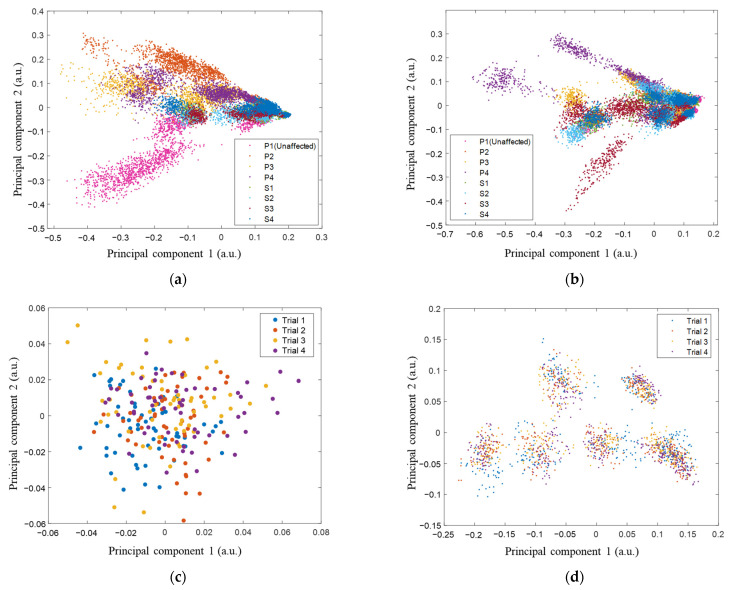
The first two principal components of feature spaces for (**a**) each level of limb position and electrode shift based on all motions combined, (**b**) each level of limb position and electrode shift based on all motions combined from another participant, (**c**) the four trials EMG in P1’s CH, and (**d**) four trials EMG in all motion combined of P1.

Each level of influencing factor was well separated as clusters represented using the first two principal components of feature spaces, where the same influencing factors were adjacent (Figure 5). The distribution of the influencing factor spread after combining all motions is shown in Figure 6a,b. As the same motion was performed, each repetition of EMG blended (Figure 6c) with no apparent clustering. When combining all motions, each four-trial feature stayed tight based on different motions’ clusters (Figure 6d).

### 3.4. Effect of Limb Position on Class Distributions on Amputees

Table 8 shows the WFC of the unaffected position and BFC accuracies for intact-limb and amputated subjects. There were no significant differences (*p* > 0.05, one-way ANOVA) in performance degradation between the intact-limb and amputated subjects.

The outcomes of the factor classification are presented in Table 9. The classification accuracies for influencing factors exceeded 98% for both able-bodied individuals and amputees. Notably, there were no significant differences (*p* > 0.05, two-way ANOVA) in the performance of influencing factor classifications between different hand motions or between able-bodied subjects and amputees. And, there was no interaction (*p* > 0.05, two-way ANOVA) between hand motions and type of subject. After combining all hand motions, the classification accuracies were 80.24 ± 9.15 and 78.36 ± 10.74 for able-bodied subjects and amputees, respectively.

**Figure 7:**
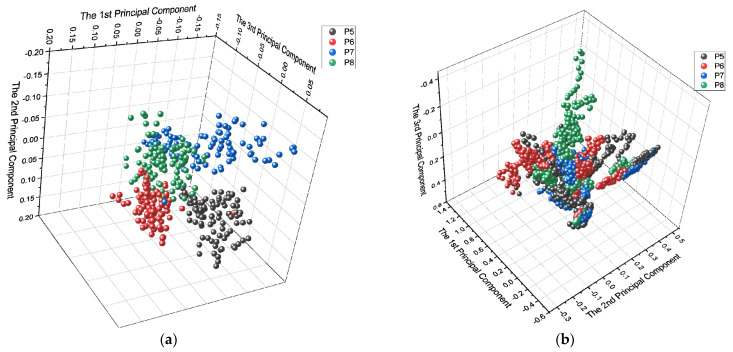
The first three principal components of feature spaces from an amputee for the influencing factor class are: (**a**) based on single motion, and (**b**) when all hand motions are combined.

Most of the feature distributions of different limb positions were separated (Figure 7a), as demonstrated previously. After combining all hand motions, all limb positions’ feature distribution overlapped (Figure 7b), resulting in worse classification accuracy.

## 4. Discussion

This study aimed to quantify how limb positions and electrode shifts affect class distributions. In motion classifications, we used LDA as it is widely used as the baseline in the research field of PR-prosthetic control and is commercialized in the prosthetic control system of COAPT [37] and OttoBock [38]. The degradation of results of inter-limb-position classifications was around 15–21%, similar to previous research [10,12,39]. There was no further decrease in accuracy in comparing five and sixteen limb position effects, but forearm orientation had a more significant impact than other limb positions [10]. In addition, previous studies [15,40] have stated that electrodes shifted along the direction of the muscle fibers have a lesser decrease in accuracy than transversal shift and accuracy decreased more with increased distance. Our results demonstrated that electrode shifts affected the classification accuracy, but there was no significant difference between different levels of electrode shifts. The average accuracy of transversal shift (S2 and S4) was slightly lower than longitudinal (S1 and S3) when the shift distances were the same. The reason could be that the displacements induced in our study were too small compared to other studies to reflect the difference, as EMG signals vary more when the electrode is near the innervation zone [14]. Nevertheless, this shift was enough to induce new class instances. 

Despite investigating the effect of combined factors [27,28,30] on the overall classification, classifying different influencing factors has not been attempted to the best of our knowledge. LDA can also be selected to classify factors. Its simplicity and fast processing time allow for the potential use of complex algorithms that depend on the results of factor identification. This enables adaptive classification to run within the limited time frame required for real-time control. Our results indicate that influence factors (limb positions vs. electrode shifts) are classifiable with high accuracy. The classification performance of the influencing factors based on every single motion (Type 1–2) with two selected classifiers and feature sets supports our hypothesis. Nevertheless, the motion did not affect the degree of classification. Upper extremity muscles exhibit similar synergistic activation patterns during the same hand movements [41].

Additionally, changes in limb position can cause alterations in muscle synergy recruitment due to factors such as gravity and variations in proprioception [42]. The variation in EMG signals induced by electrode shifts depends on the direction and magnitude of the shift. Consequently, when electrode shifts or changes in limb position occur during EMG recordings, new class instances are generated based on the different types and levels of muscle patterns. 

In the feature space, the RI was low when it was measured from the same motion within the same factor. After inducing different levels of limb positions and electrode shifts, the consistency of the same motion became lower, indicating the potential existence of factor clusters. Changes in mSI support our classification results of poor performance (due to poor separability) when many factors are combined. 

To demonstrate the existence of new class instances, we also classified motion trials (repetitions), and the results revealed accuracies below chance. This is further shown in Figure 5 and Figure 6, where factors are well separated graphically, but repetitions are not. From the perspective of feature distribution, each level of influencing factor was well separated for classification Type 1–2, where each level of the same influence factor was closely distributed. When all motions were combined (Figure 6a,b), the feature distributions of each level of influencing factor became closer. Because of the divergence of feature distribution of different motions, influencing factors were distributed in several areas in the feature space. They caused overlaps, which caused classification performance degradation subsequentially, making classification tasks challenging. 

Furthermore, in the analysis of amputees’ data, we found that the limb position did have the same extent of degradation on the performance of BFC as the able-bodied subjects. The influence factor classification performance showed no significant difference between amputees and able-bodied subjects, with promising accuracy (>98%). It demonstrated that the limb position caused by a change in feature distribution would also form corresponding class instances in amputees. The number of amputees was limited; therefore, more validation should be conducted on amputee-related datasets.

On one side, the results of this research provide a fundamental basis for designing better adaptive mechanisms that should account for the significant statistical changes in the feature space. These mechanisms should be able to separate classes when they translate into space. Dual-stage classification schemes were investigated to mitigate the effect of limb position by classifying limb position before the hand motion classification. Previous studies [43,44] stated that classifying accelerometer data can identify limb positions with promising results. However, introducing additional data could increase the potential computation cost. On the other hand, this research raises the question of the importance of recalibration when using actual prostheses. In the studies conducted by Hargrove et al. [45] and Simon et al. [5], participants recalibrated their PR-based myoelectric prostheses 32.6 ± 8.2 times and a median value 18 times with an interquartile range of 11.75 to 36 over 8 weeks home trial, respectively. Notably, the reason for recalibration was not recorded in those prior studies. It is reasonable to assume that some recalibrations were habitual, whereas others were necessitated by poor control. EMG signal quality helps in noise estimation and onset detection. If factors that cause performance degradation can be identified, it can give researchers more profound insight into the reasons for prosthetic recalibration. 

The high accuracy of influencing factor classification was achieved in the current study, showing its impact. However, the study is at an early stage, and its clinical translation for determining the reason(s) behind changes in EMG signals necessitates further investigation. Factor identification would require collecting data on different factors for training the classifier, which is time-consuming. Additionally, some within/between day factors [10] and co-existing factors may contribute to changes in the signal characteristics due to the time gap between the influencing factor data acquisition and the analysis. These issues will be addressed in future research. Moreover, as this research is limited to two factors and conducted offline, we recommend future studies where class distributions are affected in real-time with visual feedback and where the limb is in a constant state of motion. This will allow quantification of class distribution both at the initial condition when the factor occurs and while the user attempts to correct the mistake.

## 5. Conclusions

In this study, we investigated the feasibility of classifying EMG signals with different influencing factors and provide insight into the feature space distribution for each influencing factor. We have demonstrated for the first time that each factor introduces changes in the feature space that are statistically new class instances, which is directly linked to changes in RI and mSI. Thus, the feature space contains two statistically classifiable clusters when the same motion is collected in two different limb positions or electrode shifts. Our findings could pave a new path for researchers to understand the statistical changes in class distributions due to limb positions and electrode shifts and propose optimum solutions. 

## Figures and Tables

**Table 1:** Electrode shift performed in each session. All electrodes were shifted as described in the table.

Shift Number	Description
S0 (P1) *	No electrode shift
S1	Shift all 0.5 cm distally from S0.
S2	Shift all 1.0 cm distally from S0
S3	Shift all 0.5 cm transversally to the right from S0
S4	Shift all 1.0 cm transversally to the right from S0

* P1 is used as the reference electrode position as S0.

**Table 2:** Details of each type of influencing factor classification.

Classification Type	Training and Testing Data	Motions	Predict Classes
Type 1	Electrode shift and limb position data	Single motion	P1, P2, P3, P4, S1, S2, S3, S4
Type 2	Electrode shift and limb position data	Single motion	Electrode shift, Limb position
Type 3	Limb position data	All motions	P1, P2, P3, P4
Type 4	Electrode shift data	All motions	S0 (P1), S1, S2, S3, S4
Type 5	Electrode shift and limb position data	All motions	P1, P2, P3, P4, S1, S2, S3, S4

**Table 3:** Inter-factor-level of motion classification accuracy (%) under the effect of limb position when trained using P1 (unaffected) data.

	Testing Data			
	P1 (Unaffected)	P2	P3	P4
Classification Accuracy (%)	98.16 ± 5.11	85.00 ± 10.51	84.00 ± 8.61	85.99 ± 8.41

**Table 4:** Inter-factor-level of motion classification accuracy (%) under the effect of electrode shift when trained by P1 (unaffected) data.

	Testing Data				
	S0 (Unaffected)	S1	S2	S3	S4
Classification Accuracy (%)	98.16 ± 5.11	85.96 ± 13.34	83.92 ± 11.37	84.11 ± 12.88	80.89 ± 11.54

**Table 5:** Classification accuracy (%) of five types of influencing factor classification (averaged over motions).

Classification Type	Accuracy (%)
Type 1	96.13 ± 1.44
Type 2	99.05 ± 0.98
Type 3	82.27 ± 9.14
Type 4	67.03 ± 8.65
Type 5	65.40 ± 8.23

**Table 6:** Classification accuracy (%) of the first two types of influence factor classification (averaged over subjects).

Classification Type	Motions							Significant Different Motions (*p*-Value)
	CH	OH	FL	EX	PN	SN	RT	
Type 1	96.52 ± 2.34	96.97 ± 2.62	95.73 ± 4.01	94.26 ± 5.93	95.20 ± 3.71	96.51 ± 2.42	97.75 ± 1.62	0.0713
Type 2	98.62 ± 1.57	98.88 ± 1.39	98.57 ± 2.12	98.18 ± 2.90	99.55 ± 0.51	99.68 ± 0.43	99.84 ± 0.27	0.0094 *

* Significant differences only existed between RT and EX by the results of multi-comparison.

**Table 7:** Trial classification accuracy (%) within each influencing factor.

Influence Factors	P1	P2	P3	P4	S1	S2	S3	S4
Accuracy (%)	47.50 ± 3.32	48.65 ± 4.93	48.96 ± 3.15	47.20 ± 4.69	47.67 ± 3.87	48.28 ± 3.98	48.15 ± 3.77	48.96 ± 4.22

**Table 8:** Inter-factor-level of motion classification accuracy (%) under the effect of limb position when trained using P7 (unaffected) data.

	Testing Data			
	P7 (Unaffected)	P5	P6	P8
Intact-limb	99.06 ± 0.94	84.23 ± 7.18	73.37 ± 14.57	83.26 ± 7.33
Amputees	99.22 ± 0.79	75.84 ± 21.50	71.11 ± 16.99	78.83 ± 16.17

**Table 9:** Classification accuracy (%) of the Type 1 influencing factor classification (averaged over subjects).

	Motions							Significant Different Motions (*p*-Value)
	CH	OH	FL	EX	PN	SN	RT	
Able-bodied	99.96 ± 0.14	99.03 ± 1.35	99.06 ± 1.47	98.94 ± 1.57	98.64 ± 1.81	99.00 ± 1.44	99.42 ± 0.78	0.9234
Amputees	99.21 ± 1.12	98.57 ± 1.28	98.71 ± 1.81	98.85 ± 2.38	98.29 ± 1.13	99.34 ± 0.93	98.88 ± 1.59	0.2171

## Data Availability

Raw data are available for sharing if requested.
